# Long-term response of different Botulinum toxins in refractory neurogenic detrusor overactivity due to spinal cord injury

**DOI:** 10.1590/S1677-5538.IBJU.2016.0584

**Published:** 2017

**Authors:** Giuseppe Lombardi, Stefania Musco, Giovanni Bacci, Maria Celso, Valerio Bellio, Giulio Del Popolo

**Affiliations:** 1 Department of Neuro-Urology, Azienda Ospedaliero Universitaria Careggi, Firenze, Italy;; 2 Department of Biology, Universita Degli Studi di Firenze, Toscana, Italy

**Keywords:** Botulinum Toxins, Type A, Spinal Cord Compression, Urinary Bladder, Neurogenic

## Abstract

**Purpose:**

To assess the response in spinal cord injured patients alternatively treated with different types and dosages of Botulinum neurotoxin type A (BoNT/A) over 15 years.

**Material and methods:**

Patients who underwent first BoNT/A from 1999-2001 and practiced intermittent catheterization were included. Baseline 3-day bladder diary (BD) and urodynamics were collected. BoNT/A failure was defined when patients asked for re-injection ≤ 3 months post-treatment. Criteria for re-injection was at least one daily episode of urinary incontinence at BD. Before re-injection, patients were asked if they had reached 6 months of dryness without antimuscarinics (YES response).

**Results:**

Overall, 32/60 (53.4%) “No failure” (NF) group; 16 (26.6%) “occasional failure” (OF) and 12 (20%) “consecutive failure” (CF) were included. A total of 822 BoNT/A infiltrations were performed. The mean interval from previous injection to treatment re-scheduling was 8 months. No significant differences between treatments were found within the three groups (p>0.05). *T*he percentage of YES responses increased from 19% (AboBoNT/A 500IU) to 29 % (OnaBoNT/A 300IU) in NF, and from 18% (AboBoNT/A 500IU) to 25% (OnaBoNT/A 300IU) for OF. Five NF cases (15.6%) maintained 6 months of dryness after each injection. Among the baseline variables, only low compliance (< 20mL/cmH_2_O) was found as predictor for failure (p=0.006).

**Conclusions:**

Long term BoNT/A for NDO did not increase failures, independent of the types of treatments and switching. Definition of failure and other criteria for continuing repetitive BoNT/A treatment is mandatory. CF was predictable for no response in earlier follow-up.

## INTRODUCTION

One of the major health problems in patients with spinal cord injury (SCI) is bladder dysfunction. Particularly, the presence of neurogenic detrusor overactivity (NDO) may be a threat to the upper urinary tract (1-3).

In 2000, Schurch et al. first published on detrusor botulinum neurotoxin type A (BoNT/A) injections to treat NDO in SCI individuals (4). Since then, literature reports a high percentage of patients who have gained clinical and urodynamic benefits after BoNT/A, achieving urinary continence, increasing bladder capacity, reducing detrusor pressures in patients refractory to antimuscarinics (5-8). Both BoNT/A injections, Abobotulinumtoxin (Dysport®, Ipsen Biopharm, Slough, UK) as well as Onabotulinumtoxin (Botox®, Allergan, Irvine, CA, USA), have been proven to be safe with a positive impact on quality of life (9-15).

Other BoNT/A such as NT-201 (Xeomin®) or CBTX-A distributed under several different brand names in different countries (Prosigne®, Lanzox®, Lantox®, Liftox®, and Redux®) have been studied by some authors, but evidence in literature is still lower compared to the Abo and OnaBoNT/A (16).

Nevertheless, only OnaBoNT/A 200IU has been approved for NDO since 2013 in our country (17).

However, not much information is available on follow-up longer than 10 years, especially taking into account the switching of BoNT/A types (Dysport® vs. Botox® or vice versa) and/or different dosages in the same cohort of patients over time.

### Aim of the study

In this retrospective study, we report the experience of our patients affected by NDO, treated with two types of BoNT/A (Botox® and/or Dysport®) at different dosages during a 15-year follow-up.

## MATERIAL AND METHODS

Only adult SCI patients who had undergone the first BoNT/A injection for their NDO refractory and/or intolerant to antimuscarinics, and who were managing their bladder via intermittent catheterization were selected in our Italian Centre. Only NDO exclusively secondary to SCI were included. The study was conducted in accordance to the Declaration of Helsinki and the International Guidelines on Good Clinical Practice.

From December 1999 to October 2001 the first injection was administered with two different BoNT/A at various dosages: AboBoNT/A (Dysport®) 500 or 750IU and OnaBoNT/A (Botox®) 200 or 300IU.

Before the first injection and throughout the entire follow-up, a 3-day bladder diary (BD) was collected. At baseline, each subject was submitted to urodynamics as recommended by the International Continence Society (18).

The interval between the previous injection and scheduling the patient’s following injection was also recorded. We evaluated the BD at the time of rescheduling for a new injection. If unable to attend our clinic, patients were advised to send us their BD by any means of communication (e.g, fax, post and/or mail).

BoNT/A “failure” was defined as patients, within 3 months post-injection, who reported at least one daily episode of urinary incontinence in their BD.

If ineffectual, the following repeated injection was offered with the same dosage and type of BoNT/A previously used. Only after two consecutive failed attempts, a different type of BoNT/A and/or higher dosage were chosen randomly.

All those responding to the other 3 treatments were switched to OnaBoNT/A 200IU after its approval in Italy in March 2013. Those patients continued with OnaBoNT/A (Botox®) 200IU unless they had two consecutive “failures”.

The urodynamic parameters were: maximum detrusor pressure during involuntary contraction (Pdetmax), maximum cystometric capacity (MCC), and bladder compliance (Pdet at MCC).

After baseline, urodynamics pre re-injection was mandatory only when BoNT/A failure occurred.

Apart from failures, time for further urodynamic follow-ups was scheduled according to the International Guidelines depending on the individual’s risk for upper urinary tract deterioration but never exceeding 2 years (19).

Individuals taking antimuscarinics were advised to progressively stop the dosages if dryness was achieved and then start again if urinary incontinence occurred.

Prior to each new BoNT/A, the number of YES responses concerning the question on whether patients were continuously dry for at least 6 months without oral drugs, was recorded.

Subjects showing three consecutive failures were advised to undergo major surgery. For those subjects, data were reported from the first baseline up to the last injection. No BoNT/A treatment was ever performed before 3 months had elapsed from previous injections.

Detrusor infiltrations were performed on 20-30 sites, trigone and bladder neck sparing, with a 5mm 23 gauche needle and a rigid cystoscope only by experienced urologists.

The following baseline predictable variables were taken into account: ≥ 40 years old; SCI >3 years; traumatic aetiology; gender; tetraplegia; complete lesion; concomitant antimuscarinics; compliance <20mL/cmH_2_O; urinary incontinence episodes ≥ 4 per day and mean bladder capacity > 250mL at the BD.

### Statistical analysis

Statistical analyses were performed using the R software [R Core Team (2016)]. In particular, Kruskal-Wallis one-way analysis of variance was carried out to test differences in the duration of the efficacy in the three groups of patients defined, regardless of the treatment used (kruskal function of the R package ‘agricolae’ version 1.2). Similarly, differences in duration of efficacy across the four treatments here considered were tested for each group of patients. Moreover, the efficacy of the four treatments was evaluated using Linear Mixed-Effects Models with random intercept and the log-likelihood function (lme function of the ‘nlme’ R package version 3.1).

The predictable variables were assessed using a Multinomial Log-linear Model (multinom function of the R package ‘nnet’ version 7.3).

## RESULTS

### Patient population selection

From our database, a total of 72 SCI patients, of whom 19 female (26.4%), was initially identified. Twelve individuals (16.6%) were excluded: 4 due to missing data, 4 because of documented mixed urinary incontinence, 2 who were under 18 years old, and 2 who showed epilepsy as another neurological co-morbidity.

A total of 60 patients (83.3%) were included. Overall the SCI population had suprasacral lesion ≤ thoracic (T11) level and exclusively A or B degree lesions according to the American Spinal Injury Association Impairment Scale (AIS) (20).

All subjects reported at least 2 daily episodes of urinary incontinence in their BD at baseline. Individuals were sub-grouped in: “No Failure,” group (NF) who never reported BoNT/A injection “ineffectiveness” according to our criteria; “Occasional Failure” (OF) who had at least one, but not successive failures; “Consecutive Failure” (CF) subjects with persistent BoNT/A ineffectiveness.

### No failure group

**Figure 1:**
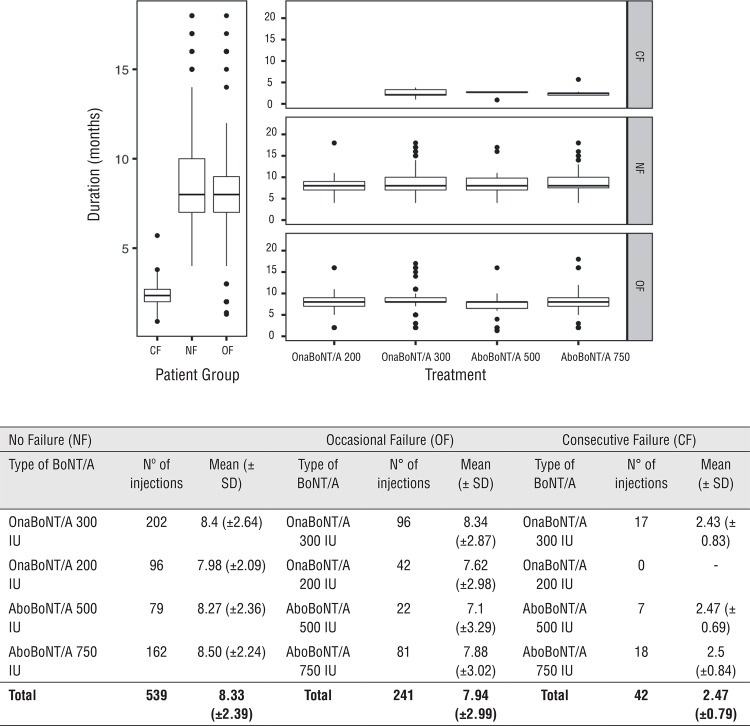
Boxplots reporting the efficacy of treatments in the three groups of patients (left panel), and across all treatments used (right panel).

Thirty-two out of 60 SCL patients (53.3%) were defined as No Failure (NF). The mean duration of efficacy in months for each BoNT/A treatment is reported in Figure-1.

**Figure 2:**
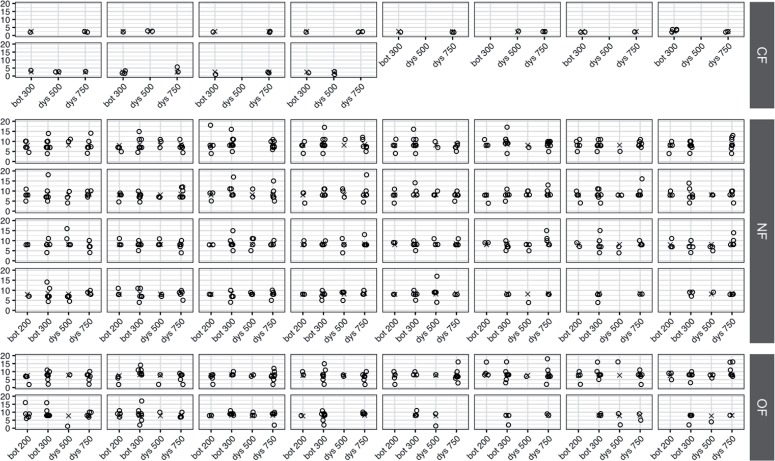
Random intercept models for each patient included in the study (The x-axis reports the different treatment used in the study whereas the y-axis reports the duration of the treatment expressed in month. Crosses report the predicted efficacy for each treatment and for each patient, computed by using linear mixed-effects models).

Twenty-seven out of 32 subjects (84.4 %) underwent all four treatments at least once (Figure-2). Twenty-nine individuals (90.6%) were still on follow-up at the last visit, while 3 patients (9.3%) had stopped treatments because they desired a definitive solution for their NOD. All of them underwent bladder augmentation respectively after 5, 6 and 7 BoNT/A detrusor injections. At BD during follow-up, the mean bladder capacity at each catheterization ranged from 210-260mL, while mean episodes of daily incontinence varied from 2.39 to 2.96.

**CF =** Consecutive Failure; **NF =** No Failure; **OF =** Occasional Failure; **Bot =** OnaBoNT/A; **Dys =** AboBoNT/A

For any BoNT/A type of treatments, the percentage of YES responses went from 19% with aboBoNT/A 500IU to 29% with onaBoNT/A 300IU (Table-1).

**Table 1:** Six months dryness (%) achieved without antimuscarinics.

	onaBoNT/A 300 IU	aboBoNT/A 750 IU	onaBoNT/A 200 IU	aboBoNT/A 500 IU
**No Failure group**				
Total number of injections	202	162	96	79
Number of injections 6-months dry (%)	58 (29%)	44 (27%)	21 (22%)	15 (19%)
**Occasional Failure group**				
Total number of injection	96	81	42	22
Number of injection 6-months dry (%)	24 (25%)	18 (22%)	8 (19%)	4 (18%)

Only five (15.6%) patients reported at least 6 months of dryness after overall injections despite the BoNT/A used.

### Occasional failure group

Sixteen out of 60 subjects (26.6%) were defined “occasional failure” (OF). The mean duration of efficacy in months for each BoNT/A treatment is reported in Figure-1.

Five and two out of 16 subjects failed twice and three times during follow-up, respectively.

Eleven (68.7%) were treated by overall types and dosages of BoNT/A (Figure-2). Twelve out of 16 (75%) were still in follow-ups.

Two out of 16 subjects (12.5%) stopped BoNT/A injections for non-urological complications. One required a permanent indwelling catheter for an unsolved sacral sore lesion. One became unable to perform self-catheterization due to the onset of cerebrovascular disease. Instead 2 patients (12.5%) quit treatment, requiring definitive alternatives for their NDO. As for the previous group, all these patients were submitted to enterocystoplasty.

At BD during follow-up the mean bladder capacity at each catheterization ranged from 230 to 265mL, whereas mean episodes of incontinence/day varied from 2.15 to 2.79.

Twenty-five urodynamics were carried out within a three-month interval post-injection (range 7-12 weeks). Mean MCC was 230mL (range 190-280mL), while mean Pdet max was 63.24cmH_2_0 (range 35-90cmH_2_0).

Low compliance was documented in the same five subjects (31.2%), both at baseline and during follow-ups.

The percentage of YES responses ranged from18% with Dysport 500IU to 25% with Botox 300IU (Table-1).

### Consecutive failure group

Twelve out of 60 individuals (20%) stopped BoNT/A injections due to consecutive failures. Nine out of 12 subjects (75%) reported failure with the first injections. All these individuals quit the follow-up before March 2013 (max follow-up 22 months). Two subjects (12.5%) were not on antimuscarinics at baseline. Nine (75%) showed low compliance at urodynamics (Table-2).

**Table 2:** Baseline characteristics of patients.

	No Failure	Occasional failure	Consecutive failures
Total numbers of patients	32	16	12
Age in years (mean ± SD)	39.97 ± 10.79	37.62 ± 10.61	39.50 ± 9.07
Duration in months of SCL pre-first BoNT/A (mean ± SD)	41.03 ± 16.87	39.25 ± 17.32	33.83 ± 18.21
Nº with traumatic aetiology of SCL (%)	27 (84.4)	13 (81.2)	11 (91.6)
Nº of males (%)	25 (78.1)	12 (75)	8 (66.6)
Nº of tetraplegics (%)	7 (21.8)	5 (31.2)	4 (33.3)
Nº with complete SCL (%)	22 (68.7)	9 (56.2)	7 (58.3)
Nº on antimuscarinics (%)	27 (84.3)	11 (68.7)	10 (83.3)
**3-day Bladder Diary**			
Bladder capacity in mL (mean ± SD)	203.12 ± 31.87	206.87 ± 34.89	193 ± 38
Episodes of daily urinary incontinence (mean ± SD)	4.11 ± 0.75	3.83 ± 0.76	4.19 ± 0.75
**Urodynamics**			
MCC in mL (mean ± SD)	190.62 ± 28.62	168.12 ± 11.06	177.50 ± 28.30
Pdetmax in cmH_2_0 (mean ± SD)	64.40 ± 10.71	63.51 ± 12.07	66.50 ± 10.50
Compliance in mL/H_2_0 (mean ± SD)	26.19 ± 13.44	24. 74 ± 14.17	17.67 ± 20.47

**SD =** Standard deviation; **Nº =** Number of patients; **BoNT/A =** Botulinum Toxin A; **SCI =** spinal cord lesion; **Pdetmax =** maximum detrusor pressure at involuntary contraction; **MCC =** Maximum Cystometric Capacity

The mean duration of efficacy in months for each BoNT/A treatment is reported in Figure-1.

Thirty-five urodynamics were performed within 3 months following injection (range 8-12 weeks).

Mean MCC was 213.14mL (range 170-240mL), while mean Pdet max was 64.74cm H_2_0 (range 35-90cmH_2_0). The same nine patients with documented low compliance at baseline showed comparable values (<20cmH_2_0/mL) during follow-up.

The 10 patients using antimuscarinics at baseline never dropped their baseline dosage of oral treatment following each BoNT/A injection.

## Statistical results

The CF group showed a pronounced difference in the duration of efficacy of the treatments with an average two-months duration against eight-months reported for the other two groups (Kruskal-Wallis test p-value <0.01) (Figure-1). No significant differences were found in the three groups of patients regarding the type of treatments considered with an average duration of efficacy per group equal to the one reported above (Kruskal-Wallis test p-values >0.05) (Figure-1). In addition, the efficacy of treatments in the three groups of patients was assessed through Linear Mixed-Effects Models with random intercept, reporting a different regression model for each patient (Figure-2). Results showed that the efficacy of the treatments drastically changes in the three groups of patients considered, regardless of the type of treatment (p-values >0.05). Considering the predictable variables, only low compliance at baseline had a significant impact on determining consecutive failures (95% confident interval [CI] 0.53-3.14; Odd Ratio [OR] 7.27; p value=0.006).

## DISCUSSION

According to our results, long term response does not depend on the type of treatments. As a matter of fact, no significant differences in mean duration of efficacy between treatments were found in the overall sample. The mean clinical efficacy was around 8 months as reported in literature in shorter follow-ups using only one type of BoNT/A at the same or different dosages (14-17).

Moreover, the switch option does not seem a valid strategy for failed patients. In the OF, patients responded to the same type and dosage of BoNT/A that had previously failed. Moreover, considering the CF, switching was not helpful to avoid a further “failure”. Although, the number of CF patients was small, this group showed a higher percentage of low compliance at baseline urodynamics compared to others. This finding is similar to that reported in literature (15, 21) and it could explain why CF discontinued follow-up earlier and 75% of them reported failure at first injection.

Concerning the use of oral antimuscarinics, whether they are useful to extend the interval between injections is still widely debatable. As a matter of fact the main RCTs were designed without antimuscarinic wash-out (9, 11). In our study the percentage of YES responses was ≤30% in both OF and NF groups. Finazzi et al. reported a similar trend about the use of antimuscarinics during the first injection follow-up. In their prospective observational study on 105 patients, only 23.8% of patients discontinued oral therapy at 120 days, whereas almost 97% of patients were on antimuscarinics with a majority of them (52%) already having returned to oxybutinin t.i.d. within 6 months following 300IU BoNT/A treatment (22). Similarly, Alvares et al. showed that 18 out of 22 subjects (81.8%) continued to use anticholinergics to achieve continence (23).

In our sample, a high percentage of SCI patients (70.7%) has been in follow-up for longer than 15 years. Seventeen out 60 patients (12 of whom were CF) asked for bladder augmentation (24, 25).

This result raises the question whether or not treating subjects with BoNT/A for longer than 15 years is the right approach.

Nowadays, although the alternative option for treating refractory NDO is bladder augmentation, over the last 10 years other lesser invasive treatments have been proposed to selected SCI patients, such as sacral neuromodulation, which may also have potential positive effects on other concomitant pelvic dysfunctions (26). Our findings (i.e. the need for continuative use of antimuscarinics, the time duration of dryness, the variability in responsiveness and possible occurrence of occasional BoNT/A ineffectiveness) ought to be shared and discussed with patients at the time of counselling and at follow-ups. Thus, patients should be well informed from the beginning about the opportunity for alternative long term solutions which could guarantee dryness.

We are aware that our study was designed retrospectively and we could not determine whether some criteria were used to switch treatment despite response. Considering this limit, our methodology requires further explanations. Firstly, at the time of inclusion in 1999, no literature was available (4). Our policy since the beginning of our experience was exclusively to repeat the same BoNT/A when failure occurred once. This approach was chosen by the fact that we did not know the real individual response to one BoNT/A rather than another, in order to definitively exclude one treatment before moving on to the next. Although our clinical behaviour and definition of non-responsiveness (CF) could be debatable, we think it was helpful to discriminate the occasional failure vs. non-responders and give stronger reasons for recommending major surgery. Again, to that end, we always objectively documented failure through urodynamics.

## CONCLUSIONS

Long-term BoNT/A for NDO did not increase failures, independent of the types of treatments. Again, switching did not improve response (14, 27, 28). Non-responsiveness (CF) was found in short follow-ups despite switching, so patients should be promptly advised about other treatment options. The possibility of an indefinite treatment period using BoNT/A for other groups of patients does not seem impractical, but definition of long term response and/or criteria for discontinuing repetitive BoNT/A injections are urgent (29). Randomized prospective comparative studies are needed to confirm our results, especially in populations with a history of BoNT/A failures.

## References

[1] Kennelly MJ, Devoe WB (2008). Overactive bladder: pharmacologic treatments in the neurogenic population. Rev Urol.

[2] Cameron AP (2010). Pharmacologic therapy for the neurogenic bladder. Urol Clin North Am.

[3] Finney SM, Andersson KE, Gillespie JI, Stewart LH (2006). Antimuscarinic drugs in detrusor overactivity and the overactive bladder syndrome: motor or sensory actions?. BJU Int.

[4] Schurch B, Stöhrer M, Kramer G, Schmid DM, Gaul G, Hauri D (2000). Botulinum-A toxin for treating detrusor hyperreflexia in spinal cord injured patients: a new alternative to anticholinergic drugs? Preliminary results. J Urol.

[5] Gamé X, Castel-Lacanal E, Bentaleb Y, Thiry-Escudié I, De Boissezon X, Malavaud B (2008). Botulinum toxin A detrusor injections in patients with neurogenic detrusor overactivity significantly decrease the incidence of symptomatic urinary tract infections. Eur Urol.

[6] Jia C, Liao LM, Chen G, Sui Y (2013). Detrusor botulinum toxin A injection significantly decreased urinary tract infection in patients with traumatic spinal cord injury. Spinal Cord.

[7] Karsenty G, Denys P, Amarenco G, De Seze M, Gamé X, Haab F (2008). Botulinum toxin A (Botox) intradetrusor injections in adults with neurogenic detrusor overactivity/neurogenic overactive bladder: a systematic literature review. Eur Urol.

[8] Reitz A, Stöhrer M, Kramer G, Del Popolo G, Chartier-Kastler E, Pannek J (2004). European experience of 200 cases treated with botulinum-A toxin injections into the detrusor muscle for urinary incontinence due to neurogenic detrusor overactivity. Eur Urol.

[9] Sussman D, Patel V, Del Popolo G, Lam W, Globe D, Pommerville P (2013). Treatment satisfaction and improvement in health-related quality of life with onabotulinumtoxinA in patients with urinary incontinence due to neurogenic detrusor overactivity. Neurourol Urodyn.

[10] Schurch B, Denys P, Kozma CM, Reese PR, Slaton T, Barron RL (2007). Botulinum toxin A improves the quality of life of patients with neurogenic urinary incontinence. Eur Urol.

[11] Grosse J, Kramer G, Stöhrer M (2005). Success of repeat detrusor injections of botulinum a toxin in patients with severe neurogenic detrusor overactivity and incontinence. Eur Urol.

[12] Gomes CM, Castro JE, Rejowski RF, Trigo-Rocha FE, Bruschini H, Barros TE (2010). Experience with different botulinum toxins for the treatment of refractory neurogenic detrusor overactivity. Int Braz J Urol.

[13] Del Popolo G, Filocamo MT, Li Marzi V, Macchiarella A, Cecconi F, Lombardi G (2008). Neurogenic detrusor overactivity treated with english botulinum toxin a: 8-year experience of one single centre. Eur Urol.

[14] Rovner E, Dmochowski R, Chapple C, Thompson C, Lam W, Haag-Molkenteller C (2013). OnabotulinumtoxinA improves urodynamic outcomes in patients with neurogenic detrusor overactivity. Neurourol Urodyn.

[15] Stoehrer M, Wolff A, Kramer G, Steiner R, Lmöchner-Ernst D, Leuth D (2009). Treatment of neurogenic detrusor overactivity with botulinum toxin A: the first seven years. Urol Int.

[16] Gomes CM, Castro JE, Rejowski RF, Trigo-Rocha FE, Bruschini H, Barros TE (2010). Experience with different botulinum toxins for the treatment of refractory neurogenic detrusor overactivity. Int Braz J Urol.

[17] Kennelly M, Dmochowski R, Schulte-Baukloh H, Ethans K, Del Popolo G, Moore C (2017). Efficacy and safety of onabotulinumtoxinA therapy are sustained over 4 years of treatment in patients with neurogenic detrusor overactivity: Final results of a long-term extension study. Neurourol Urodyn.

[18] Schäfer W, Abrams P, Liao L, Mattiasson A, Pesce F, Spangberg A (2002). Good urodynamic practices: uroflowmetry, filling cystometry, and pressure-flow studies. Neurourol Urodyn.

[19] Blok B, Pannek J, Castro Diaz D (2015). EAU guidelines on neurourology. European Association of Urology.

[20] Maynard FM, Bracken MB, Creasey G, Ditunno JF, Donovan WH, Ducker TB (1997). International Standards for Neurological and Functional Classification of Spinal Cord Injury. American Spinal Injury Association. Spinal Cord.

[21] Klaphajone J, Kitisomprayoonkul W, Sriplakit S (2005). Botulinum toxin type A injections for treating neurogenic detrusor overactivity combined with low-compliance bladder in patients with spinal cord lesions. Arch Phys Med Rehabil.

[22] Finazzi-Agrò E, Topazio L, Perugia C, Lombardi G, Finita Celso M, De Nunzio C (2013). The use of oxybutynin in patients treated by means of botulinum neurotoxin A for neurogenic detrusor overactivity: an observational study. Spinal Cord.

[23] Alvares RA, Silva JA, Barboza AL, Monteiro RT (2010). Botulinum toxin A in the treatment of spinal cord injury patients with refractory neurogenic detrusor overactivity. Int Braz J Urol.

[24] Johnson EU, Singh G (2013). Long-term outcomes of urinary tract reconstruction in patients with neurogenic urinary tract dysfunction. Indian J Urol.

[25] Martens FM, Heesakkers JP (2011). Clinical results of a brindley procedure: sacral anterior root stimulation in combination with a rhizotomy of the dorsal roots. Adv Urol.

[26] Lombardi G, Nelli F, Mencarini M, Del Popolo G (2011). Clinical concomitant benefits on pelvic floor dysfunctions after sacral neuromodulation in patients with incomplete spinal cord injury. Spinal Cord.

[27] Mehta S, Hill D, McIntyre A, Foley N, Hsieh J, Ethans K (2013). Meta-analysis of botulinum toxin A detrusor injections in the treatment of neurogenic detrusor overactivity after spinal cord injury. Arch Phys Med Rehabil.

[28] Grise P, Ruffion A, Denys P, Egon G, Chartier Kastler E (2010). Efficacy and tolerability of botulinum toxin type A in patients with neurogenic detrusor overactivity and without concomitant anticholinergic therapy: comparison of two doses. Eur Urol.

[29] Soljanik I (2013). Efficacy and safety of botulinum toxin A intradetrusor injections in adults with neurogenic detrusor overactivity/neurogenic overactive bladder: a systematic review. Drugs.

